# Rapid MALDI-TOF MS identification of commercial truffles

**DOI:** 10.1038/s41598-019-54214-x

**Published:** 2019-11-27

**Authors:** Khalid El Karkouri, Carine Couderc, Philippe Decloquement, Annick Abeille, Didier Raoult

**Affiliations:** 1 IHU-Méditerranée Infection, Marseille, France; 2 Aix Marseille Univ, IRD, AP-HM, SSA, VITROME, Marseille, France; 3 Aix Marseille Univ, IRD, AP-HM, MEPHI, Marseille, France

**Keywords:** Mass spectrometry, Fungi

## Abstract

Truffles are edible mushrooms with similar morphological characteristics, that make it difficult to distinguish between highly prized truffles (such as the Périgord black *T*. *melanosporum*) and inexpensive truffles (such as the Asian Black *T*. *indicum*). These biological and economic features have led to several misidentifications and/or fraudulent profit in the truffle markets. In this paper, we investigate Matrix-assisted Laser Desorption/Ionization Time-Of-Flight Mass Spectrometry (MALDI-TOF MS) biotyping to identify 34 commercial fresh truffles from Europe and Asia. The MALDI-TOF MS clustering rapidly distinguished seven *Tuber* species identified by ITS phylogenetic analysis. The tasty *T*. *melanosporum* was clearly differentiated from the Chinese and less expensive truffles. These cheaper mushrooms were marketed as *T*. *indicum* but corresponded to a mix of three species. In total, the method confirmed misidentifications in 26% of commercial specimens. Several unknown blind-coded truffles were rapidly identified, with scores >= 2, using the Bruker Biotyper algorithm against MS databases. This study demonstrates that MALDI-TOF MS is a reliable, rapid and cheaper new tool compared with molecular methods for the identification of truffle species and could be used to control frauds in the truffle markets. It could also be useful for the certification of truffle-inoculated seedlings and/or diversity in forest ecosystems.

## Introduction

Truffles are Ascomycetes belonging to the genus *Tuber* F. H. Wigg. (order: Tuberacaea, family: Pezizales). These edible mushrooms are hypogenous and form ectomycorrhizae, a mutualistic symbiosis with the ascocarps (i.e. the truffles) being produced by the roots of shrubs and trees. The Alba white truffle (*Tuber magnatum* Pico) and the Périgord black truffle (*Tuber melanosporum* Vittad.) are some of the most sought-after and expensive species that dominate truffle markets worldwide (2018 prices in France were 3,000–5,000 euros/kg and 700–1,200 euros/kg respectively)^1–4^. They have great ecological, organoleptic (i.e., taste and aroma) and socio-economic value but, at the same time, they are morphologically similar and evolutionary related to other less sought-after truffles, such as the European white truffles, *T*. *maculatum* and *T*. *borchii*^5,6^ and the Asian black truffles of the *T*. *indicum* species complex^7,8^. Truffles of either *T*. *magnatum* or *T*. *melanosporum* can, therefore, be morphologically mistaken for, or accidentally mixed with, other less tasty truffles. Since around 1993, exports of *T*. *indicum* truffles from China to Europe have increased dramatically^7^ leading to mixed ascocarps of truffle species that might be sold as *T*. *melanosporum*^9,10^. Some truffle species, such as *T*. *indicum* have been described as being highly competitive, have a broad host spectrum and are capable of establishing symbioses outside their native range, indicating some ecological traits of invasive species^11^. Indeed, truffle trees believed to have been inoculated with *T*. *melanosporum* exhibited ectomycorrhizas or ascocarps of *T*. *indicum* or other truffle species in Oregon, USA and New Zealand^11,12^. Similarly, roots thought to be colonised by *T*. *magnatum* were found to be associated with or have produced other *Tuber* species, including *T*. *borchii* and/or *T*. *maculatum*^13^.

Thus, both the organoleptic and economic values, as well as the ecological role of truffles, have led to the development of molecular tools in order to (i) accurately identify truffle species at important stages in their life cycles (*i*.*e*., as ectomycorhizae or ascocarps), (ii) clarify the taxonomy and evolution of the genus *Tuber*, (iii) control the commercial fraud in truffle markets and agroindustrial foods, and (iv) certify the quality of truffle-inoculated seedlings in nurseries for a large-scale production^2,13–16^. The molecular markers and methods that have been most widely used to distinguish the prized *Tuber* species are the PCR-RFLP, species-specific primers, barcoding and phylogeny of the internal transcribed spacer (ITS) of the nuclear ribosomal RNA (rRNA) repeat unit, and the *β-*tubulin gene^5,7,8,10,13,15–23^. However, although these conventional DNA-based methods are more powerful than limited morphological-based techniques, they are still time-consuming, expensive and difficult to use for large-scale truffle identification.

In the last decade, the Matrix-assisted Laser Desorption/Ionization Time-Of-Flight Mass Spectrometry (MALDI-TOF MS) method has emerged as not only a powerful identification tool but also as a cost-effective and a faster technique compared to PCR based-methods^24,25^. This easier technique has been routinely used for the identification and classification of microorganisms^26–30^, fungi^31–35^, microbiota^36^ and vectors (including ticks and mosquitoes)^37–39^ in a variety of fields, such as clinical diagnosis and environmental and food monitoring^40^. To our knowledge, MALDI-TOF MS has not yet been used to identify edible mushrooms, such as truffles. In this study, we focused our investigation on MALDI-TOF MS biotyping to reliably and rapidly distinguish and identify commercial truffles from Europe and Asia after checking their identities using phylogenetic analysis of ITS sequences.

## Results

### Phylogeny of ITS sequences

**Figure 1:**
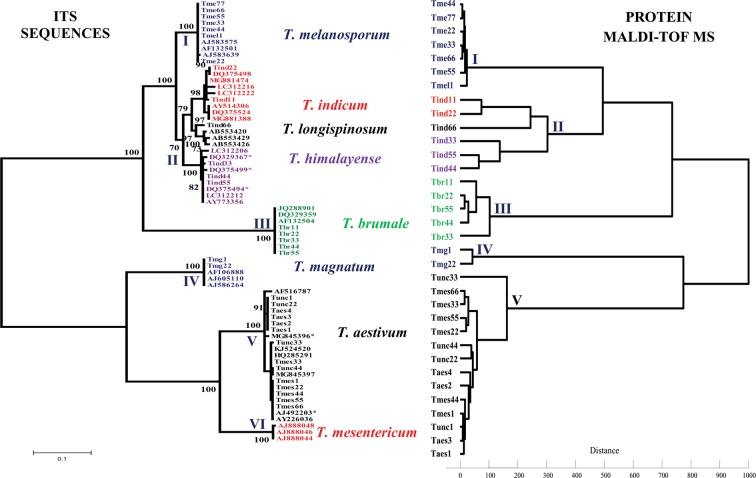
Phylogenetic tree and dendrogram inferred from ITS sequences and protein MALDI-TOF MS of *Tuber* species, respectively. The “*” symbol means that the specimens were misannotated as *T*. *indicum* or *T*. *uncinatum* in the GenBank database.

The phylogenetic tree of the 34 commercial truffles and their best BLAST homologs (Table 1) displayed six major clades that we refer to here from I to VI (Fig. 1).

**Table 1:** List of the 34 commercial fresh truffles identified by BLAST homology of ITS sequences.

Commercial identification	Specimen reference	Origin	Season	Best BLAST match
*T*. *melanosporum*	Tmel1	Australia	Summer	*T*. *melanosporum* Vitt.
	Tme22	France	Winter	
	Tme33			
	Tme44			
	Tme55			
	Tme66			
	Tme77			
*T*. *indicum*	Tind11	China	Winter	*T*. *indicum* Cooke et M.
	Tind22			
	Tind33			
	Tind44			
	Tind55			
	Tind66		Winter	*T*. *longispinosum* A. K.
*T*. *brumale*	Tbr11	France	Winter	*T*. *brumale* Vitt.
	Tbr22			
	Tbr33			
	Tbr44			
	Tbr55			
*T*. *aestivum*	Taes1	France/Spain	Summer	*T*. *aestivum* Vitt.
	Taes2			
	Taes3			
	Taes4			
*T*. *uncinatum*	Tunc1	Bulgaria	Autumn	*T*. *aestivum* Vitt.
	Tunc22			
	Tunc33			
	Tunc44			
*T*. *mesentericum*	Tmes1	France	Autumn	*T*. *aestivum* Vitt.
	Tmes22			
	Tmes33			
	Tmes44			
	Tmes55			
	Tmes66			
*T*. *magnatum*	Tmg1	France	Autumn	*T*. *magnatum* Pico
	Tmg22

The seven truffles marketed as *T*. *melanosporum* from France and Australia clustered into the firmly supported monophyletic clade I (BP = 100%), with their best homologs (BLAST sequence identities ids = 99–100%) belonging to *T*. *melanosporum* reference specimens from Europe. Similarly, the five French truffles marketed as *T*. *brumale* formed the individual clade III (BP = 100%) with their best European specimens belonging to the same species (ids = 99–100%). These European black truffles, *T*. *melanosporum* and *T*. *brumale*, were clearly distinguished from the Asian black truffles which clustered into the comprehensively supported clade II from China and Japan (BP = 70%). Interestingly, this clade divided the six commercial *T*. *indicum* truffles (“Tind“ specimens) and their best homologs (ids = 97–99% excluding misidentified/misannotated ITSs in GenBank database) into three well-supported sister subclades (BP = 97–100%) corresponding to three previously defined lineages: *T*. *indicum* (BP = 98%), *T*. *longispinosum* (BP = 97%), and *T*. *himalayense* (BP = 100%). An examination of the sequence homology between *T*. *indicum* (Tind11/22), *T*. *longispinosum* (Tind66) and *T*. *himalayense* (Tind33/44/55) specimens revealed sequence identities ranging from 92 to 95%. Of the six Chinese truffles marketed as *T*. *indicum*, 67% were found to have been misidentified by phylogenetic analysis.

The two white truffles marketed as *T*. *magnatum* from France (“Tmg” specimens) fall into the firmly supported and monophyletic clade IV (BP = 100%), with their reference specimens of the same species from Europe (ids = 99–100%). The 13 European black truffles marketed as *T*. *aestivum* from France or Spain (four “Taes” specimens), *T*. *uncinatum* from Bulgaria (four “Tunc” specimens) and *T*. *mesentericum* from France (five “Tmes” specimens) formed the large individual clade V with high branch support (BP = 100%), mostly with *T*. *aestivum* and rarely with *T*. *uncinatum* specimens from Europe (ids = 99–100%). This analysis showed that all 13 truffles belong to the same species *T*. *aestivum* from which truffles marketed as *T*. *aestivum* were collected in summer, while those marketed as *T*. *uncinatum* or misidentified as *T*. *mesentericum* were collected in autumn. All 13 *T*. *aestivum* truffles were clearly distinguished from those of their close relatives belonging to *T*. *magnatum* (clade IV) and *T*. *mesentericum* (clade VI). The latter species formed the significant and monophyletic clade VI (BP = 100%) including reference specimens from the GenBank database and none from the 34 examined truffles.

In total, the phylogenetic tree made it possible to distinguish and identify seven *Tuber* species among the 34 commercial truffles: *T*. *melanosporum*, *T*. *indicum*, *T*. *longispinosum*, *T*. *himalayense*, *T*. *brumale*, *T*. *aestivum* and *T*. *magnatum*. It also confirmed the identity of 72% of the 34 truffles marketed from Europe and China and revealed that the remaining 26% were incorrectly identified.

### Clustering MALDI-TOF MS

**Figure 2:**
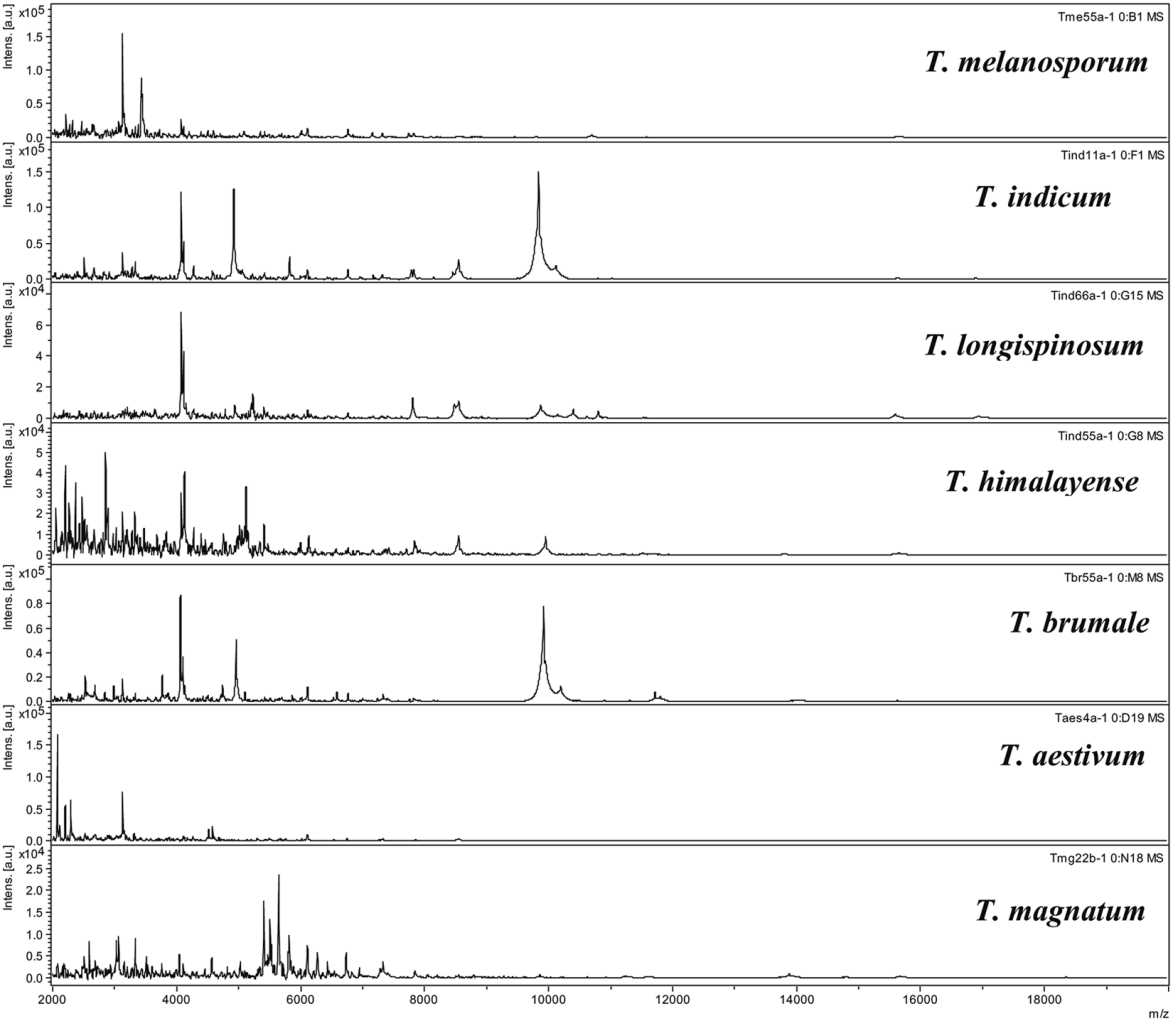
MALDI-TOF mass spectral profiles of the seven *Tuber* species identified in this study. Intens. [a.u.]: Intensity [arbitrary unit].

The protein MALDI-TOF MS analysis of the 34 commercial truffles belonging to the seven *Tuber* species identified by ITS phylogeny yielded a total of 204 MS spectra. Most of the identified peaks were found to be highly reproducible, exhibiting strong signal intensities between the six biological and technical replicates of each truffle (see one example in Supplementary Fig. S1). The MALDI-TOF MS dendrogram of the 34 commercial truffles exhibited a topology similar to the one obtained with the phylogenetic tree of ITS sequences (Fig. 1). It distinguished the seven *Tuber* species into seven clusters and sub-clusters, indicating that they harboured distinct MS profiles (Figs. 1 and 2). Clusters I and III brought together all black *T*. *melanosporum* truffles from France and Australia and *T*. *brumale* from France, respectively. Cluster II distinguished all the Chinese black truffles from the European black truffles *T*. *melanosporum* and *T*. *brumale*. This cluster consisted of three sub clusters distinguishing the three Chinese truffle species: *T*. *indicum* Tind11/22, *T*. *longispinosum* Tind66 and *T*. *himalayense* Tind33/44/55. Cluster IV differentiated all white truffles of *T*. *magnatum* species collected in France from all their close relatives in cluster V and belonging to *T*. *aestivum* species from Bulgaria, France or Spain. Overall, the MALDI-TOF MS dendrogram also confirmed that nine (26% of the 34) commercial truffles marketed as *T*. *indicum* or *T*. *mesentericum* species were misidentified.

### Blind test of MALDI-TOF MS

**Figure 3:**
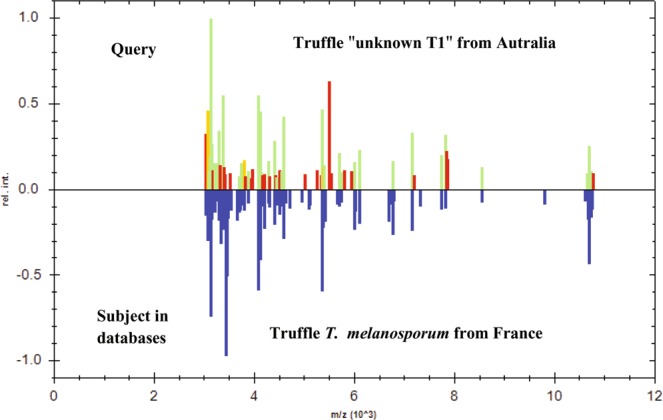
Rapid identification of the blind-coded truffle from Australia using the MALDI Biotyper search algorithm against databases containing reference MS spectra of archaea, bacteria and eukaryota including truffles. The first best hit corresponds to the MS spectrum of *T*. *melanosporum* species from France (score 2.41).

To better assess the robustness of the MALDI-TOF MS for the identification of unknown truffles, we performed a real-time blind test using a naive operator as follows: MS spectra of ten truffles coded as “unknown T*” (as queries) were subjected to a spectral homology search using the Bruker Biotyper algorithm against in-house and Bruker databases containing 7,346 reference MS spectra of bacteria, archaea and eukaryotes including truffles (Supplementary Table S1). Two (out of ten) coded truffles “unknown T1 and T2”, belonging to *T*. *melanosporum* species and collected from Australia and France, respectively, were rapidly and unequivocally identified as *T*. *melanosporum* species. Both specimens displayed best matches with six truffles of the same species (with scores >= 2.12). Figure 3 shows the first best match diagram with high MS peak similarities with the “unknown T1 truffle”, which belongs to *T*. *melanosporum* species from Australia, and the *T*. *melanosporum* species from France. Similarly, the six other truffles coded as “unknown T3/4, T5/6/7 and T8” from Europe, were also efficiently identified by best matching MS profiles to their corresponding species,*T*. *brumale*, *T*. *aestivum* and *T*. *magnatum* from Europe, respectively, with scores of between 2 and 2.66. Similarly, the two Chinese truffles coded as “unknown T9 and T10” and belonging to *T*. *indicum* and *T*. *himalayense* first best matched *T*. *indicum* and *T*. *himalayense* (with scores of 2.28 and 2.41), respectively. Of all the unknown truffles examined, only the Chinese truffle coded as “unknown T10” exhibited a second best match with a slightly lower score of 1.83 with another strain of the same species *T*. *himalayense*. Moreover, the *T*. *indicum* truffle coded as “unknown T9” displayed a lower score of 1.78 with its closely related species *T*. *himalayense*.

All Biotyper matches for the ten unknown truffles with scores below 1.7 were not considered reliable identifications as defined by the MALDI Biotyper software.

## Discussion

In this study, the MALDI-TOF MS method was evaluated with ITS phylogeny for the identification of 34 commercial truffles from Europe and Asia.

The MALDI-TOF MS dendrogram clearly distinguishes seven *Tuber* species among the 34 truffles identified by phylogenetic analysis. Morphologically similar and phylogenetically related truffle species that have distinct organoleptic and socio-economic value were easily differentiated. For instance, the highly prized *T*. *melanosporum* truffles (European black) were distinguished from those belonging to the *T*. *indicum* species complex and *T*. *brumale* species (Chinese and European black truffles), which are less economically significant in Europe. Of the Chinese truffles, MALDI-TOF MS distinguished three species: *T*. *indicum*, *T*. *longispinosum* and *T*. *himalayense*. This finding is in agreement with the taxonomy of the so-called *T*. *indicum* species complex (Asian black truffle) in highly discussed reports using phylogenetic, phylogeographic and/or population genetic investigations^8,20,21,41^. Indeed, truffles in this complex exhibit several morphologically similar and evolutionary related features, making it difficult to delimit the species. Moreover, three phylogenetic lineages have been described among the *T*. *indicum* complex and correspond to the two distinct and sympatrically distributed lineages, *T*. *indicum* and *T*. *himalayense* species from China^7,8,41,42^. Finally, a recent study in Japan identified a new monophyletic species named *T*. *longispinosum* A. Kinosh sp. nov., which is a sister to the *T*. *indicum* species^23^. To our knowledge, this is the first study reporting a *T*. *longispinosum* truffle in China. Our findings support the presence of these three lineages within the complex *T*. *indicum* in Asia and thus improve its taxonomy and distribution.

In this study, all truffles of a same species clustered together into a single cluster or sub-cluster indicating that they have conserved MS profiles and peaks, which are not influenced by their distinct or similar geographical origin and/or season, as well as their intraspecific variability. For example, the seven *T*. *melanosporum* truffles that were collected in the summer and autumn in Australia and France, respectively, were found together into the cluster I. In another example, the 13*T*. *aestivum* truffles that were collected in the summer or autumn in Bulgaria, France or Spain as *T*. *aestivum* or *T*. *uncinatum*, formed the single cluster V. Fierce debate on the taxonomy of *T*. *aestivum* and *T*. *uncinatum* has been reported^43–47^. However, our findings are in agreement with molecular studies that named *T*. *aestivum* and *T*. *uncinatum* as the Burgundy truffle *T*. *aestivum* (syn. *uncinatum*), a species which is considered as the most common European truffle and was discovered in many habitats over Europe; their fruitbodies ripening from late-May until winter^15,43,44^. Moreover, our results corroborate other molecular phylogenetic studies that have shown that they are conspecific^44–46^ and suggest that ecological rather than genetic causes may explain differences in sporal morphology, taste and smell between truffles of these species^47^.

The MALDI-TOF MS biotyping used in this study also confirmed that 26% of the 34 commercial truffles examined were misidentified in truffle markets, as revealed by the phylogenetic analysis. Indeed, six and three truffles marketed under the name of *T*. *mesentericum* from France and *T*. *indicum* from China respectively were found to belong to *T*. *aestivum* and *T*. *longispinosum* or *T*. *himaleyense*. This may be due not only to the accidental mixing of morphologically similar truffle species at the time of their harvest by hunters and traders who continue to enjoy attractive economic benefits^41,48–50^, but also to the lack or absence of any reliable control and certification before their arrival on the markets^5,10,14,51^.

Like BLAST homology search algorithms, the Bruker Biotyper search algorithm is used for the identification of unknown specimens after a spectral homology search with MS profiles in reference databases of bacteria, archaea and eukaryotes. In this study, the Biotyper tool allowed the unequivocal and rapid identification of ten unknown truffles belonging to *T*. *melanosporum*, *T*. *brumale*, *T*. *aestivum* (syn. *uncinatum*), *T*. *magnatum*, *T*. *indicum* and *T*. *himalayense*. These results were obtained with best matches scores higher than 2, a value which is in agreement with the defined Biotyper scores for species identification. However, among the Chinese truffles, the unknown *T*. *himalayense* truffle exhibited a second best match with the same species, with a score of 1.83, while the unknown *T*. *indicum* truffle displayed a second best match with its closely related species *T*. *himalayense*, with a slightly lower score of 1.78. Although these scores were not consistent with the defined Biotyper criteria for microbial species and interspecies identification, they suggest that further MALDI-TOF MS spectra of diverse Chinese truffles are needed to enrich our truffle database and to define their comprehensive MS score cut-offs at the species and closely related species levels. Indeed, a recent clinical microbiological study suggested that the reduction of the species cut-off threshold from 2.0 to 1.7 significantly increased the identification rates of Gram-negative rods and *Aspergillus* species^26,34^.

Several recent studies have shown that truffle glebae contain various microbial communities as shown in 16S-metagenomic or metaproteomic studies^52–55^. However, in our investigation, none of the unknown truffles examined by the Bruker Biotyper best matched with scores >= 1.7 of the 7,366 reference spectra of bacteria, archaea and other eukaryotes. This indicates that most of the identified MS peaks belong to fungal truffle species, and that bacteria and/or other biological organisms present in the gleba were not sufficiently abundant to be detected.

## Conclusion

To the best of our knowledge, this is the first time that the MALDI-TOF MS biotyping has been applied to the identification of truffle species. It enables a rapid and reliable distinction and identification of species as well as the detection of misidentified specimens among commercial truffles. This technique, together with the phylogenetic tree, provides new insights into the taxonomy of the complex *T*. *indicum* and confirmed that *T*. *aestivum* and *T*. *uncinatum* are conspecific.

The MS acquisition and identification of truffle protein extracts was rapidly and easily performed in less than 30 minutes, a shorter time than that used with PCR amplification and sequencing methods. Therefore, the development of such routine and less expensive approach may provide an efficient tool for experts in the truffle markets and truffle diversity analysis in research and environmental projects. Furthermore, its application to the certification of large-scale inoculated truffle seedlings and quality control of truffle products should be examined. This may lead to better control and traceability that could contribute towards the protection of truffle resources and increased diversity from overexploitation of ecological habitats.

## Methods

### DNA extraction, PCR and sequencing

A total of 34 commercial fresh truffles marketed as *T. melanosporum* (n = 7 ascocarps), *T. indicum* (n = 6 asco.), *T. brumale* (n = 5 asco.), *T. aestivum* (n = 4 asco.), *T. uncinatum* (n = 4 asco.), *T. mesentericum* (n = 5 asco.) and *T. magnatum* (n = 2 asco.) were obtained in the summer, autumn and winter (2017/2018) from markets of truffles from France, Bulgaria, Spain, Australia and China (Table 1). Total genomic DNA was extracted from 30 mg of each ascocarp gleba using the EZ1 DNA Tissue Kit (Qiagen, Courtaboeuf, France), according to the manufacturer’s instructions. DNA quantification was performed using the Qubit 4 Fluorometer (Invitrogen, Thermo Fisher Scientific). The nuclear rDNA internal transcribed spacer ITS (ITS1 + 5.8 S + ITS2) was amplified with the universal ITS1 and ITS4 primer set^56^. The PCR mixture was set up in a reaction volume of 25 µL as follows: 5 µL template DNA (0.5 ng µL^−1^), 0.75 µL of each primer (10 pmol µL^−1^), 12.5 µL AmpliTaq Gold PCR Mix (Life Technologies) and 6 µL of ddH_2_O. PCR amplification was performed using the Applied Biosystems 2720 Thermal Cycler. Cycling conditions were set as follows: initial denaturation at 95 °C for 15 minutes, 39 cycles of denaturation at 95 °C for 30 seconds, annealing at 55 °C for 30 seconds and amplification at 72 °C for one minute, and a final extension at 72 °C for five minutes. Negative controls (no template DNA) were also performed. PCR products were run on 1% agarose gel to check their amplification and sizes. Both DNA strands of amplicons were determined with the original primers and then sequenced using the BigDye Terminator Cycle Sequencing Kit on an AB 3130xl Genetic Analyser Sequencer. ITS sequences were edited using the DNAbaser assembler v4.36 and deposited in the GenBank database under the accession numbers MN104000-33.

### Phylogenetic analysis

To check the identity of each commercial truffle, ITS sequences were subjected to BLASTn searches against the GenBank database (National Center for Biotechnology Information) and to phylogenetic analysis. For each ITS truffle, we downloaded between five and ten best homolog sequences corresponding to reference and/or voucher specimens from GenBank database^7,8,19,22,23,42^. Multiple ITS sequence alignments of marketed and reference truffles were carried out using ClustalW implemented in the BioEdit software, with default parameters. Redundant sequences from GenBank databases and ambiguously aligned sites were visually inspected and excluded before phylogenetic inference. The maximum likelihood (ML) phylogenetic tree was performed with the software MEGA v7.0^57^ under the K2P + I model^58^, the Nearest-Neighbor-Interchange (NNI) heuristic search and the partial deletion. Node robustness was estimated through bootstrap (BP) analysis of 1,000 replicates^59^. BP values that were lower than 70% were removed from the tree.

### Sample preparation for MALDI-TOF MS

Sample extractions and analysis were performed as described by Yssouf *et al*. and Flaudrops *et al*.^27,60^. Two biological replicates cut from each truffle gleba were used for protein extraction. Each piece (8–10 mg) was mixed with 400 μL of 70% formic acid (Sigma, Lyon, France), 400 μL of 100% acetonitrile (VWR Prolabo) and ground with acid washed glass beads (Sigma Aldrich, Lyon France) in a polypropylene tube using the FAST Prep®−24 Instrument (MP Biomedicals, Illkirch-Graffenstaden, France). The homogenates were centrifuged for two minutes at 13,000 × *g* and, 1.5 μL of each supernatant was spotted onto a steel MALDI target plate (Bruker Daltonics, Wissembourg, France) in triplicate and then dried at room temperature for 15 minutes. 1.5 μL of a CHCA matrix suspension (saturated α-Cyano-4-hydroxy-cinnamic acid, 50% acetonitrile, 2.5% trifluoroacetic acid and HPLC water) (Sigma) was then overlaid onto each spot to allow crystallisation. To control loading on the plate, matrix quality and the MALDI-TOF apparatus performance, 1.5 μL of the matrix solution was loaded in triplicate onto the plate with (positive control) or without (negative control) Bruker Protein Calibration Standard I. Finally, the plate was dried at room temperature for 15 minutes and was then immediately introduced into the Autoflex-Speed linear MALDI-TOF MS for analysis (Bruker Daltonics, Germany).

### MS acquisition and processing

Protein mass profiles were acquired using FlexControl 3.4 software (Bruker Daltonics, Germany). The spectra were recorded in the linear positive-ion mode (ion source 1 = 19.5 kV, ion source 2 = 18.2 kV, detector voltage = 2872 V, pulsed ion extraction = 200 ns) with a mass range from 2 to 20 kDa^27,60^. Each spectrum corresponded to ion accumulation of 10,000 laser shots randomly distributed on the spot. The spectra obtained were processed with default parameters [Savitsky-Golay smoothing (5 Da, two runs), top hat algorithm for baseline subtractions, peak picking (150 maximum, S/N >= 3), peak width = 5 m/z] using FlexAnalysis v.3.4 software (Bruker Daltonics, Germany).

### MS clustering and real-time blind testing

To assess the reproducibility of the truffle spectra, MS profiles of the six replicates of each truffle were aligned and compared using FlexAnalysis software. The Main Spectra Projection (MSP) of the six reproducible spectra of each truffle in each species was computed with standard parameters (mass range = 3–15 kDa, maximum mass error tolerance for each spectrum = 2,000 ppm, mass error for the MSP = 200 ppm, minimum peak frequency = 25%, maximum number of peaks = 70) using the Bruker Biotyper software v3.0. To test the potential of MALDI-TOF MS for the distinction of truffle species, a dendrogram comparing the MSP spectra was constructed using the following parameters implemented using the same tool: hierarchical clustering algorithm, distance method = correlation, linkage = average. To further test the reliability of MALDI-TOF MS for the identification of truffles, a real-time blind test was carried out between unknown truffles (as queries) and four MS reference databases (as subjects), by a naive operator, using the Bruker Biotyper search algorithm. This algorithm yielded best match scores which reflect the degree of homology between queries and references, making it possible to identify (or not) the unknown specimens. The MS spectra of ten truffles coded as “unknown T1 to T10” and belonging to five different species were compared to the Bruker databases containing 7,346 reference MS spectra of bacteria, archaea and eukaryotes, as well as to an in-house comprehensive database including MS spectra of reference truffle species. For each unknown specimen, MS spectra of six biological and technical replicates were not available in the databases when searching for best matches using the Biotyper software.

## Supplementary information

Dataset 1
