# Supplementary material for: Rapid MALDI-TOF MS identification of commercial truffles
Source: Sci Rep. 2019 Nov 27;9:17686. doi: 10.1038/s41598-019-54214-x (PMC6881316; doi:10.1038/s41598-019-54214-x)
Supplement: Supplementary file 1 — Dataset 1 [file 41598_2019_54214_MOESM1_ESM.pdf]

Supplementary Information

Rapid MALDI-TOF MS identification of commercial truffles

Khalid El Karkouri, Carine Couderc, Philippe Decloquement, Annick Abeille and Didier Raoult

Supplementary Figure S1. Truffle specimen "Tbr55" exhibiting highly reproducible MS profiles corresponding to its six biological and technical replicates.

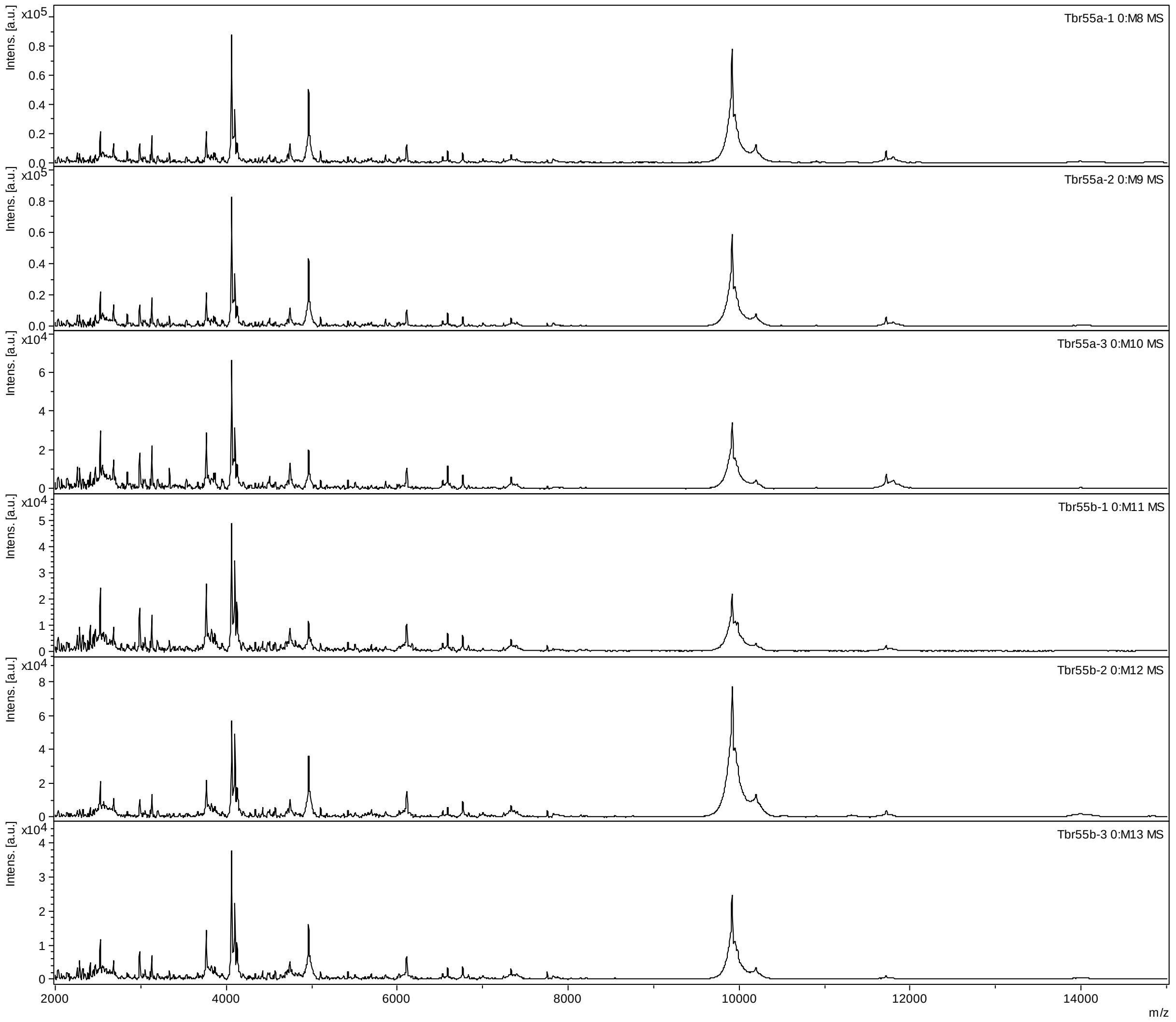

Supplementary Table S1. Identification of unknown truffles by searching spectral homology against MS reference databases using the MALDI Biotyper software. The first eight best hits are presented for each query. Green = score between 2 and 3, species identification; yellow = score between 1,7 and 1.999, closely related species or distant strains of the same species; Red = score between 0 and 1,699, identification not reliable.

Query = truffle "unknown T1" (*T. melanosporum* from Australia)

**Rank (Quality)**	**Matched Pattern**	**Score Value**
1 (+++)	Tuber melanosporum Tme44	2.41
2 (+++)	Tuber melanosporum Tme33	2.41
3 (+++)	Tuber melanosporum Tme55	2.4
4 (+++)	Tuber melanosporum Tme22	2.35
5 (+++)	Tuber melanosporum Tme66	2.24
6 (++)	Tuber melanosporum Tme77	2.12
7 (-)	Tuber indicum Tind11	1.47
8 (-)	Tuber indicum Tind55	1.37

Query = truffle "unknown T2" (*T. melanosporum* from France)

**Rank (Quality)**	**Matched Pattern**	**Score Value**
1 (+++)	Tuber melanosporum Tme33	2.66
2 (+++)	Tuber melanosporum Tme44	2.6
3 (+++)	Tuber melanosporum Tme66	2.53
4 (+++)	Tuber melanosporum Tme22	2.52
5 (+++)	Tuber melanosporum Tmel1	2.4
6 (+++)	Tuber melanosporum Tme77	2.38
7 (+)	Tuber indicum Tind11	1.81
8 (-)	Tuber indicum Tind33	1.59

Query = truffle "unknown T3" (*T. brumale* from France)

Rank (Quality)	Matched Pattern	Score Value
1 (+++)	Tuber brumale Tbr22	2.57
2 (+++)	Tuber brumale Tbr44	2.55
3 (+++)	Tuber brumale Tbr11	2.43
4 (++)	Tuber brumale Tbr33	2.2
5 (-)	Tuber melanosporum Tme66	1.29
6 (-)	Tuber melanosporum Tme77	1.26
7 (-)	Tuber melanosporum Tme55	1.24
8 (-)	Tuber melanosporum Tme44	1.23

Query = truffle "unknown T4" (*T. brumale* from France)

Rank (Quality)	Matched Pattern	Score Value
1 (+++)	Tuber brumale Tbr55	2.58
2 (+++)	Tuber brumale Tbr11	2.47
3 (+++)	Tuber brumale Tbr44	2.4
4 (+++)	Tuber brumale Tbr33	2.33
5 (-)	Tuber melanosporum Tme66	1.66
6 (-)	Tuber melanosporum Tme55	1.51
7 (-)	Tuber melanosporum Tme22	1.5
8 (-)	Tuber indicum Tind11	1.48

Query = truffle "unknown T5" [*T. aestivum* (syn. *T. uncinatum*) from France]

Rank (Quality)	Matched Pattern	Score Value
1 (+++)	Tuber aestivum (syn. T. uncinatum) Taes3	2.43
2 (+++)	Tuber aestivum (syn. T. uncinatum) Taes4	2.4
3 (+++)	Tuber aestivum (syn. T. uncinatum) Taes1	2.4
4 (++)	Tuber aestivum (syn. T. uncinatum) Tmes1	2.24
5 (++)	Tuber aestivum (syn. T. uncinatum) Tunc1	2.23
6 (++)	Tuber aestivum (syn. T. uncinatum) Tmes44	2.15
7 (++)	Tuber aestivum (syn. T. uncinatum) Tunc44	2.13
8 (++)	Tuber aestivum (syn. T. uncinatum) Tmes66	2.07

Query = truffle "unknown T6" [*T. aestivum* (syn. *T. uncinatum*) from Bulgaria]

Rank (Quality)	Matched Pattern	Score Value
1 (+++)	Tuber aestivum (syn. T. uncinatum) Tunc44	2.3
2 (++)	TTuber aestivum (syn. T. uncinatum) Tmes44	2.29
3 (++)	Tuber aestivum (syn. T. uncinatum) Tmes22	2.27
4 (++)	Tuber aestivum (syn. T. uncinatum) Tmes55	2.24
5 (++)	Tuber aestivum (syn. T. uncinatum) Tunc1	2.23
6 (++)	Tuber aestivum (syn. T. uncinatum) Tmes1	2.22
7 (++)	Tuber aestivum (syn. T. uncinatum) Taes3	2.2
8 (++)	Tuber aestivum (syn. T. uncinatum) Taes4	2.13

Query = truffle “unknown T7” [*T. aestivum* (syn. *T. uncinatum*) from France]

Rank (Quality)	Matched Pattern	Score Value
1 (+++)	Tuber aestivum (syn. T. uncinatum) Tmes1	2.61
2 (+++)	Tuber aestivum (syn. T. uncinatum) Tmes22	2.43
3 (+++)	Tuber aestivum (syn. T. uncinatum) Tunc1	2.34
4 (+++)	Tuber aestivum (syn. T. uncinatum)Taes3	2.27
4 (++)	Tuber aestivum (syn. T. uncinatum) Tunc22	2.26
6 (++)	Tuber aestivum (syn. T. uncinatum)) Tunc44	2.23
7 (++)	Tuber aestivum (syn. T. uncinatum) Tmes55	2.21
8 (++)	Tuber aestivum (syn. T. uncinatum) Taes4	2.17

Query = truffle “unknown T8” (*T. magnatum* from France)

Rank (Quality)	Matched Pattern	Score Value
1 (+++)	Tuber magnatum Tmg22	2.39
2 (-)	Tuber brumale Tbr22	1.47
3 (-)	Candida valida DSM 70169 DSM	1.35
4 (-)	Candida guilliermondii CBS 566 CBS	1.27
5 (-)	Tuber aestivum/uncinatum Tunc44	1.27
6 (-)	Lactobacillus plantarum ssp plantarum DSM 20174T DSM	1.23
7 (-)	Clostridium chauvoei 1024_NCTC 8596 BOG	1.21
8 (-)	Tuber aestivum (syn. T. uncinatum) Taes3	1.18

Query = truffle “unknown T9” (*T. indicum* from China)

Rank (Quality)	Matched Pattern	Score Value
1 (++)	Tuber indicum Tind11	2.28
2 (+)	Tuber himalayense Tind55	1.78
3 (-)	Tuber himalayense Tind44	1.6
4 (-)	Tuber longispinosum Tind66	1.6
5 (-)	Tuber melanosporum Tme44	1.6
6 (-)	Tuber melanosporum Tme77	1.58
7 (-)	Tuber melanosporum Tme33	1.54
8 (-)	Tuber melanosporum Tme55	1.44

Query = truffle “unknown T10” (*T. himalayense*from China)

Rank (Quality)	Matched Pattern	Score Value
1 (+++)	Tuber himalayense Tind55	2.41
2 (+)	Tuber himalayense Tind33	1.83
3 (-)	Tuber longispinosum Tind66	1.64
4 (-)	Tuber indicum Tind22	1.61
5 (-)	Candida albicans CBS 2312 CBS	1.4
6 (-)	Filifactor villosus 1051_NCTC 11220T BOG	1.35
7 (-)	Tuber melanosporum Tme77	1.34
8 (-)	Sphingomonas sp B605 UFL	1.34
